# Microbial associations of shallow-water Mediterranean marine cave Solenogastres (Mollusca)

**DOI:** 10.7717/peerj.12655

**Published:** 2021-12-15

**Authors:** Elena Vortsepneva, Pierre Chevaldonné, Alexandra Klyukina, Elizaveta Naduvaeva, Christiane Todt, Anna Zhadan, Alexander Tzetlin, Ilya Kublanov

**Affiliations:** 1 Invertebrate Zoology Department, Biological Faculty, M. V. Lomonosov Moscow State University, Moscow, Russia; 2 IMBE, CNRS, Aix Marseille University, IRD, Avignon University, Station Marine d’Endoume, Marseille, France; 3 Winogradsky Institute of Microbiology, Research Center of Biotechnology of the Russian Academy of Sciences, Moscow, Russia; 4 Rådgivende Biologer AS, Bergen, Norway; 5 White Sea Biological Station, Biological Faculty, M. V. Lomonosov Moscow State University, Moscow, Russia

**Keywords:** Microbial symbionts, Solenogastres, Marine cave, Thaumarchaeota, NGS

## Abstract

The first cave-dwelling Solenogastres—marine shell-less worm-like mollusks—were sampled from Mediterranean marine caves floor silt in the Marseille area. The mollusks were 1.5 mm in length, had a transparent body with shiny spicules and appear to represent a new *Tegulaherpia* species. Electron microscopy revealed a high number of microbial cells, located on the surface of the spicules as well as in the cuticle of *Tegulaherpia* sp. The observed microbial cells varied in morphology and were unequally distributed through the cuticle, reaching a highest density on the dorsal and lateral sides and being practically absent on the ventral side. Next Generation Sequencing (NGS) of V4 region of 16S rRNA gene amplicons, obtained from the DNA samples of whole bodies of *Tegulaherpia* sp. revealed three dominating microorganisms, two of which were bacteria of Bacteroidetes and Nitrospirae phyla, while the third one represented archaea of Thaumarchaeota phylum. The Operational Taxonomic Unit (OTU), affiliated with Bacteroidetes was an uncultured bacteria of the family *Saprospiraceae* (93–95% of Bacteroidetes and 25–44% of the total community, depending on sample), OTU, affiliated with Nitrospirae belonged to the genus *Nitrospira* (8–30% of the community), while the thaumarchaeal OTU was classified as *Candidatus* Nitrosopumilus (11–15% of the community). Members of these three microbial taxa are known to form associations with various marine animals such as sponges or snails where they contribute to nitrogen metabolism or the decomposition of biopolymers. A similar role is assumed to be played by the microorganisms associated with *Tegulaherpia* sp.

## Introduction

Although the northwestern Mediterranean marine biota is one of the best-studied in the world, some remote, less-accessible ecosystems are still likely hotspots of unknown diversity. Underwater marine caves are numerous in the Marseille area (SE France) due to the karstic nature of the seashore that cannot be accessed by standard oceanographic gear and can only be explored by speleo-divers (Harmelin, Vacelet & Vasseur, 1985). Due to the darkness and low water circulation, these caves are characterized by oligotrophy, in a very similar way to the deep sea. As a consequence, some of the microorganisms from marine caves are also deepwater species, or species belonging to taxa for which deepwater representatives are known (Calado, Chevaldonné & Santos, 2004; Bakran-Petricioli et al., 2007; Janssen, Chevaldonné & Martinez-Arbizu, 2013; Chevaldonné et al., 2015; Cárdenas et al., 2018; Chevaldonné & Pretus, 2021). Moreover, some exceptional caves further provide a cold thermal regime (mean ca. 13–15 °C), similar to that of the Mediterranean deep sea areas (Vacelet, Boury-Esnault & Harmelin, 1994; Bakran-Petricioli et al., 2007).

Our knowledge of the benthic communities of marine caves is still incomplete. Most emphasis has been devoted to the most conspicuous components of either the fixed fauna, such as sponges or bryozoans (*e.g.,* Harmelin, Vacelet & Vasseur, 1985; Harmelin, 1997; Grenier et al., 2018), or the mobile fauna such as teleost fish and crustaceans (e.g., Ledoyer, 1989; Chevaldonné & Lejeusne, 2003; Bussotti, Di Franco A. Francour & Guidetti, 2015). Some studies have focused on cave sediment meiofauna, often with an emphasis on targeted taxonomic groups (see examples in Janssen, Chevaldonné & Martinez-Arbizu, 2013; Zeppilli et al., 2018). Meiofauna proved to be a good indicator of ecological processes in marine caves. Near Marseille, Janssen, Chevaldonné & Martinez-Arbizu (2013) studied the meiofauna of the 3PP marine cave (depth 25–30 m below sea level) sediment, one of such exceptional cold-water caves. The 3PP cave was characterized by very low abundances of meiofaunal organisms usually found at abyssal sites (Janssen, Chevaldonné & Martinez-Arbizu, 2013). Moreover, they noted significant differences in meiofauna community along the transect from the outside to the innermost part of the cave: tardigrades were restricted to the inner parts of the cave, while copepod diversity decreased towards the inner parts. Such interesting findings prompted further investigations of the macro- and meiofauna, especially of yet poorly-studied groups.

Although Sørensen, Jørgensen & Boesgaard (2000) and Boesgaard & Kristensen (2001) pointed to the presence of unknown aplacophoran species in one cave system in Australia, there has been no mention of Solenogastres in marine cave studies so far. Solenogastres (Mollusca) is a small group of marine shell-less worm-like mollusks that inhabit various depths, from the sublittoral to the abyssal, including hydrothermal vents (Scheltema, 2008). They are mostly epibenthic or epizoic organisms living and feeding on cnidarians, while some groups feed on other organisms such as polychaetes, nemerteans, and bryozoans (Todt & Salvini-Plawen, 2005; Salvini-Plawen & Oztürk, 2006; García-Álvarez & Salvini-Plawen, 2007; Bergmeier, Ostermair & Jörger, 2021). Most species are less than 5 mm in length with the smallest being less than a millimeter long and the biggest reaching over 300 mm (Todt, 2013). One of the significant morphological characteristics of Solenogastres is the integument with a thick cuticle composed of a glycoprotein complex with high concentration of acid mucopolysaccharides, low concentrations of protein (Beedham & Trueman, 1968) and chitin (Furuhashi et al., 2009). The cuticle of Solenogastres contains calcareous spicules of different shapes, originated from the epidermal epithelium (Scheltema, Tscherkassky & Kuzirian, 1994). The surface of most Solenogastres remains clean from bacteria, but microbial associations have been found in three Solenogastres species including *Neomenia carinata* from soft bottoms at 18–565 m depth (Scheltema, Tscherkassky & Kuzirian, 1994) and two species from hydrothermal vents, *Helicoradomenia* cf. *acredema* and *Helicoradomenia* sp. (Katz, Cavanaugh & Bright, 2006). The bacterial symbionts of the two hot vent *Helicoradomenia* species had similar morphology and epi- and endocuticular localization (Katz, Cavanaugh & Bright, 2006). Since the epidermis of Solenogastres contains secretory cells (Scheltema, Tscherkassky & Kuzirian, 1994), it is possible that the epicuticular bacteria can obtain energy or/and nutrients from the secreted compounds (Katz, Cavanaugh & Bright, 2006). In its turn, endocuticular bacteria might be chitinolytic, as it is known for many marine heterotrophic bacteria (Cottrell et al., 2000).

The present work was aimed to investigate the diversity of microbial community associated with a new species of Solenogastres, living in marine caves in the area of Marseille (NW Mediterranean Sea). The possible Sonegostres-microorganisms interactions are also discussed.

## Material and Methods

All material was sampled in the Calanques National Park, near Marseille, in the middle (ca. 40 m from entrance) and the deep (ca. 60 m from entrance) parts of Jarre cave (17 m depth, 43°11′45′N, 5°22′55′E). Bottom sediment was collected by SCUBA diving with a 20 cm-wide box on ca. one cm sediment depth and a length of 120 cm. In May 2019, 1 specimen was obtained from the middle part and 3 from the deep part of the cave, while in October 2019, 2 were found in the middle and 2 in the deep part. Five specimens were used for morphological studies (two for SEM and three for TEM) and three specimens fixed in 96% ethanol for molecular studies, of the latter one specimen was collected in May and two in October.

### Morphological studies

All studies specimens of *Tegulaherpia* sp. were relaxed before fixation using isotonic to seawater magnesium chloride solution. For transmission electron microscopy, three specimens were fixed in 2.5% glutaraldehyde (Electron Microscopy Supplies (EMS, Pennsylvania, USA) and post-fixed in 1% osmium tetroxide (EMS, Pennsylvania, USA) buffered with 0.1 mol sodium cacodylate buffer. Following steps, dehydration and embedding to the Spurr resin (EMS, Pennsylvania, USA), were performed according to Vortsepneva, Herbert & Kantor (2021).

Ultrathin (70–80 nm) sections were made using a diamond knife (Diatome, Jumbo) and Leica EM UC6 and UC7 ultramicrotomes. All ultrathin sections were contrasted using 1% uranyl acetate and 0.4% lead citrate according the protocol (Vortsepneva, Herbert & Kantor, 2021). Ultrathin sections were examined using a Jeol JEM 1011 transmission electron microscope.

For scanning electron microscopy (SEM) two specimens were fixed using the same protocol as for the TEM, followed by dehydrating and drying as it was performed by (Vortsepneva, Herbert & Kantor, 2021). The specimens were mounted on aluminum stubs, sputter coated with platinum and palladium, and examined using JEOL JSM-6380L (JEOL, USA) and CamScan S2 (Cambridge Instrument Scientific Company, England) scanning electron microscopes.

### DNA isolation and sequencing

Three specimens were fixed in 96% ethanol and stored at −20 °C for four to six months. The whole bodies of three *Tegulaherpia* sp. specimens were used for the study of their microbial communities. Total DNA was isolated with DNeasy PowerLyzer Microbial Kit (Qiagen, Germany) according to the manufacturer’s instructions using FastPrep-24™ 5G bead beating grinder and lysis system (MP Biomedicals, USA). The concentration of isolated DNA was measured using Qubit™ dsDNA HS Assay Kit (Thermo Fisher Scientific, USA) and Qubit 2.0 fluorimeter (Thermo Fisher Scientific, USA). Purified DNA was stored at −20 °C.

Amplicon libraries were prepared as described in Gohl et al. (2016). Two consecutive rounds of PCR were performed on a StepOne Plus Real-Time instrument (Thermo Fisher Scientific, USA) using qPCRmix-HS SYBR mixture (Evrogen, Russia). The primers for the V4 region of 16S rRNA gene (Fadrosh et al., 2014) contained the Illumina TruSeq sequencing primer adapters and 515F/Pro-mod-805R primer sequences (Hugerth et al., 2014; Merkel et al., 2019) were used for the first amplification step: forward primer (5′-TCG TCG GCA GCG TCA GAT GTG TAT AAG AGA CAG NNN NNN GTG BCA GCM GCC GCG GTA A-3′), and reverse primer (5′- GTC TCG TGG GCT CGG AGA TGT GTA TAA GAG ACA GNN NNN NGA CTA CNV GGG TMT CTA ATC C-3′). The first PCR amplification was performed as follows: 32 cycles of denaturation at 95 °C for 25 s; primer annealing at 56 °C for 20 s; DNA synthesis at 72 °C for 30 s, and a final elongation at 72 °C for 20 min. The second PCR stage was performed using barcoding primers as described by Gohl et al. (2016). The second amplification was performed as follows: 10 cycles of denaturation at 95 °C for 20 s, primer annealing at 59 °C for 20 s, DNA synthesis at 72 °C, for 30 s, and a final elongation step at 72 °C for 20 min. The resulting PCR products were used for the preparation of libraries for Illumina sequencing.

High-throughput sequencing of the libraries was performed with MiSeq Reagent Micro Kit v2 (300-cycles) MS-103-1002 (Illumina, USA) on a MiSeq sequencer (Illumina, USA) according to the manufacturer’s instructions.

The raw reads were processed as described in Gavrilov et al. (2019). All the reads of the V4 region of 16S rRNA gene obtained in two replicates for each sample were analyzed using the SILVAngs service with default parameters (https://ngs.arb-silva.de/silvangs/) and SILVA138.1 SSU database. NCBI BLASTn (https://blast.ncbi.nlm.nih.gov/Blast.cgi) with various parameters, and databases were used for manual curation of taxonomy of the sequences of interest. The current version of the SILVAngs (as of August 2021) uses the Silva taxonomy that is, in turn, based on the Genome Taxonomy Database (GTDB, https://gtdb.ecogenomic.org/) taxonomy. However, since this taxonomy is still not generally accepted and unfamiliar to a wide range of readers we will use the taxa approved by the Bergey’s Manual of Systematics of Archaea and Bacteria (https://onlinelibrary.wiley.com/doi/book/10.1002/9781118960608) indicating the correspondence of taxa in these two taxonomical systems in the results section.

All the obtained sequences were deposited into the NCBI under BioProject accession number PRJNA773997.

## Results

### External morphology and identification

**Figure 1:**
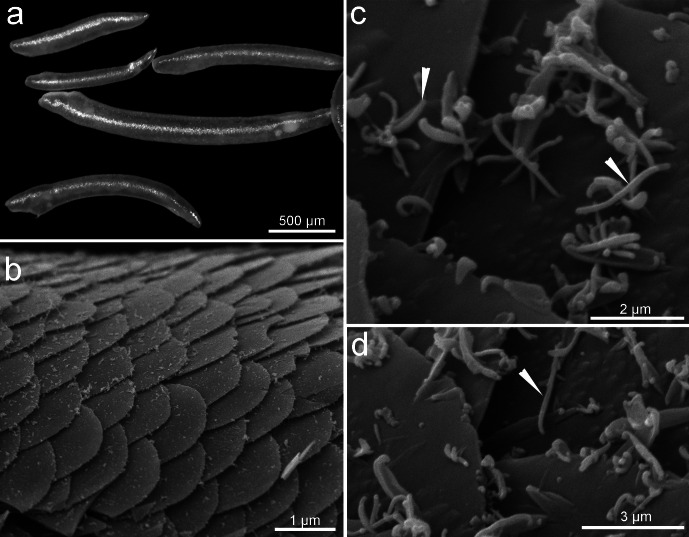
General morphology of sales of *Tegulaherpia sp*. (A) Photo light microscopy, (B–D) SEM. a. General view of different in size specimens. b. View from above of the body surface. (C–D) Scales with bacteria on the scales surface. Head arrows labeled bacterial cells.

The length of the Solenogastres specimens collected for this work varied from one to two mm. The body was light-colored and covered by scales (Figs. 1A, 1B), which gave a characteristic shine. The specimens had uniform scale-like sclerites, a distichous radula, lateroventral foregut glands belonging to type A (with ducts and extraepithlial gland cells), copulatory spicules, and the mouth opening separated from the vestibular cavity. These morphological characteristics (Salvini-Plawen, 1986; García-Álvarez & Salvini-Plawen, 2007) allow us to propose a novel Solenogastres species, belonging to the *Tegulaherpia* genus.

### Integument morphology and microbial cells

Two major morphotypes of microbial cells occurred on the surface of the body, identified with scanning electron microscopy as rods and cocci (Figs. 1B, 1C). Bacterial cells were present at the anterior end and absent at the posterior end of the mollusks (Figs. 1B, 1C).

**Figure 2:**
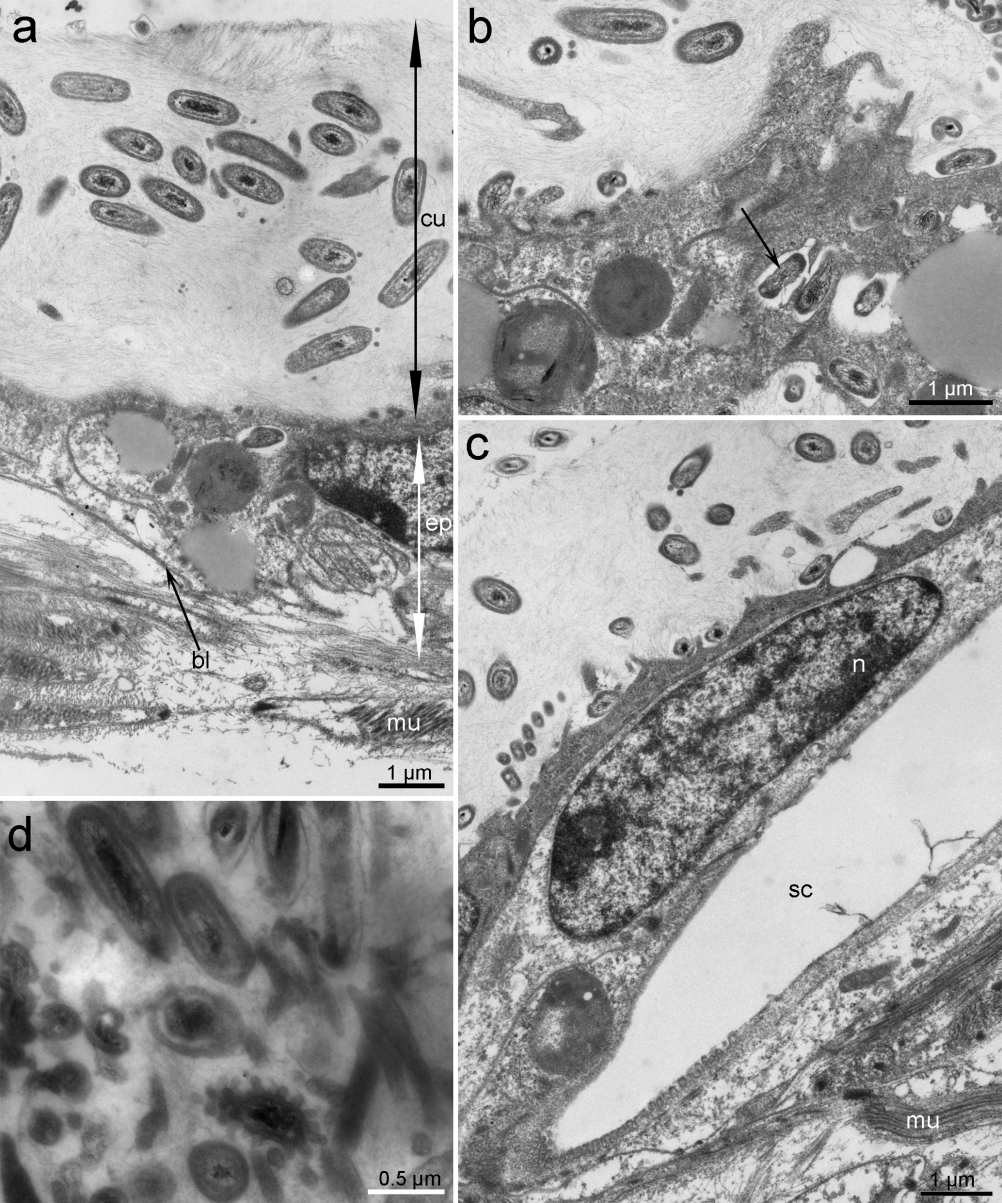
Ultrastructure of the epithelium and cuticle of *Tegulaherpia sp*. Transversal section, TEM micrographs. (A) Epithelium and cuticle containing bacteria. (B) Apical part of epithelium and bacteria immersed in epithelial cells (arrow). (C) Epithelium with a hole from forming scale. (D) Cuticle with different morphotypes of bacteria. bl, basal lamina; ep, epithelium; cu, cuticle; mu, muscles; n, nucleus; sc, scale.

**Figure 3:**
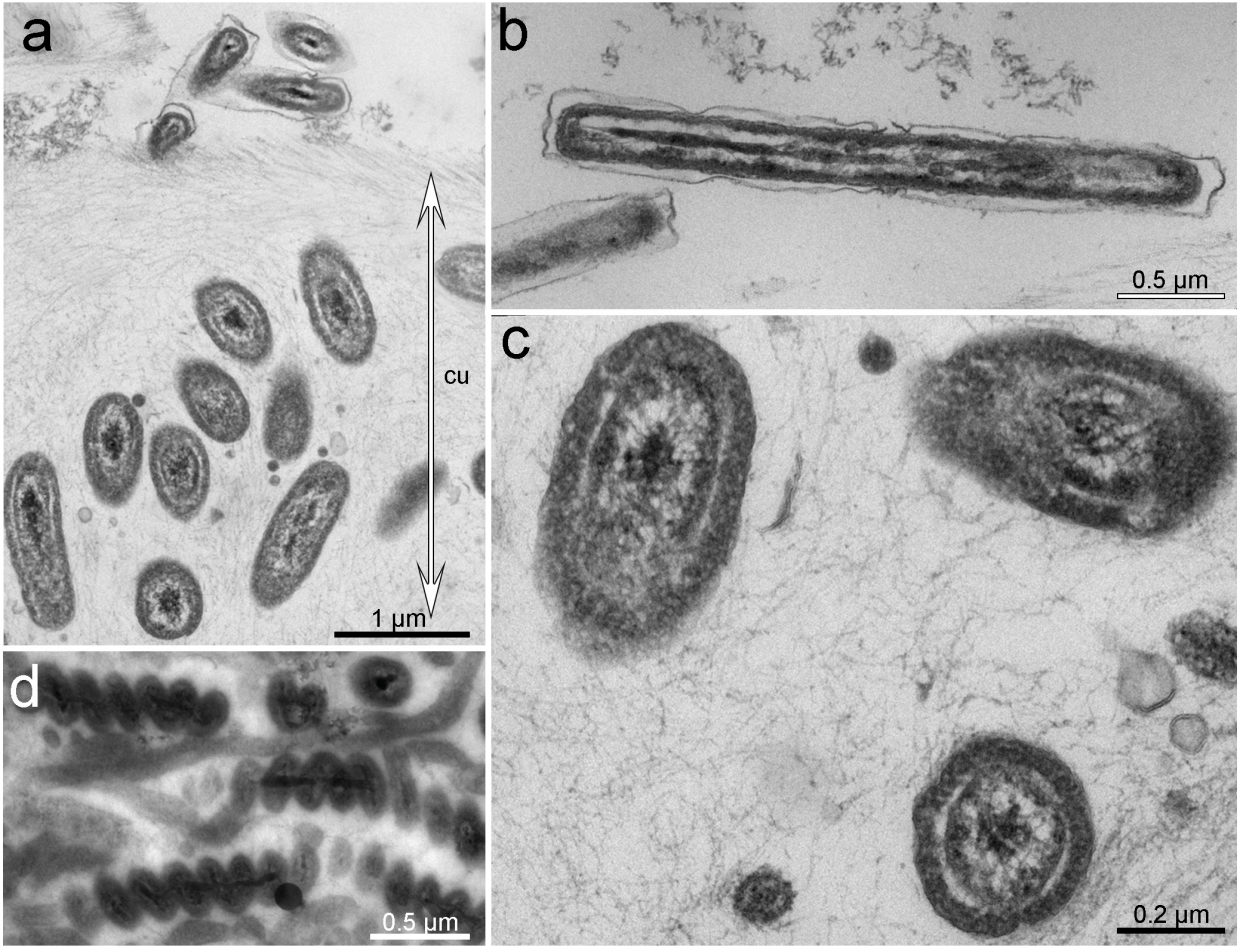
Ultrastructure of bacteria associated with cuticle. TEM micrographs. (A) Cuticle with rods inside and at the upper border of the cuticle. (B) Bacteria with Gram-negative cell-wall type at the upper layer of the cuticle. (C) Rod-shaped or cocci bacteria located within the cuticle. (D) Spirilla, located within the cuticle.

The study of ultrathin sections of the cuticle, which was 6 µm thick, revealed that the prokaryotes are not only located on the mollusks’ surface but also within the deeper layers of the cuticle of the anterior part (Fig. 2). The microorganisms were distributed unequally through the cuticle: they reached a relatively high density on the dorsal and lateral sides and were practically absent on the ventral side (Fig. 3).

Based on TEM, four main morphotypes of prokaryotes associated with *Tegulaherpia* sp. were identified: short rods (Figs. 2, 3A, 3C), long rods (Fig. 3B), spirilla-like (Fig. 3D), and cocci (Fig. 2D). Short rods were located inside the cuticle and also appeared to enter the epithelial cells of the mollusk (Fig. 3B). Long rods were only located on the surface of the cuticle (Fig. 3B). This type, as well as cocci, was the most numerous cells, found on the cuticle surface. The average length of the spirilla was 2 µm, and they were located exclusively inside the cuticle. Cocci (1 µm) were less common and were not found in the epithelium.

### Microbial community composition

**Figure 4:**
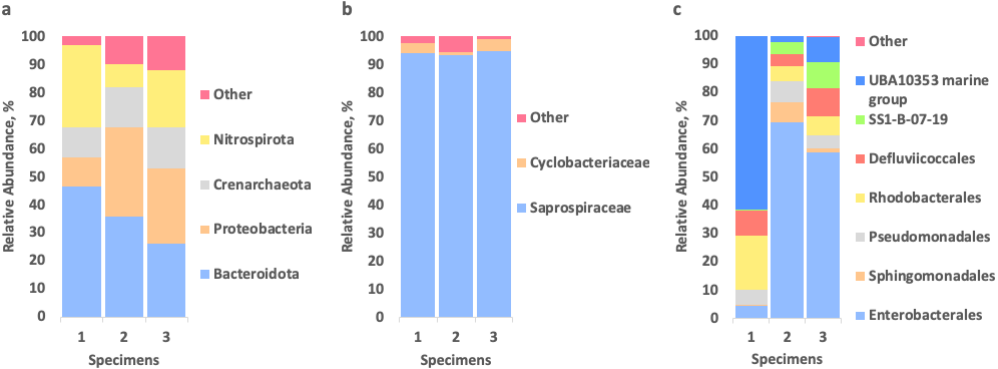
Relative abundance of prokaryotes, associated with three specimens of *Tegulaherpia sp.* according to the 16S rRNA gene sequence (V4 region) analysis. (A) Total community, phylum level; (B) Bacteroidetes (= Bacteroidota in Silva/GTDB) distribution on the level of families; (C) Proteobacteria distribution on the level of orders. Nitrospirae (= Nitrospirota in Silva/GTDB) and Thaumarchaeota (= Crenarchaeota/Nitrososphaeria in Silva/GTDB) were represented by single OTUs (see the text).

To identify the microbial community associated with *Tegulaherpia* sp., the amplicons of the V4 region of the 16S rRNA (SSU) genes were sequenced from the total DNA, isolated from the whole bodies of three individual mollusks. The representatives of bacterial phyla Bacteroidetes (26–47%), Proteobacteria (10–32%), Nitrospirae (8–30%), as well as of the archaeal phylum Thaumarchaeota (11–15%), predominated in all samples (Fig. 4).

Bacteroidetes were represented almost exclusively by an uncultured bacterium of the family *Saprospiraceae* (93–95% of Bacteroidetes and 25–44% of the total community). Results of manual BLAST of this most abundant OTU, belonging to *Saprospiraceae*, using the nr/nt database with “type material” limitation resulted in *Haliscomenobacter* and *Lewinella* members among the best hits (≤89% of sequence identities with the query). Nitrospirae were represented solely by one OTU, belonging to the genus *Nitrospira*, which is a second after *Saprospiraceae* most abundant OTU (8–30% depending on the specimen of *Tegulaherpia* sp). The classes *Alphaproteobacteria* and *Gammaproteobacteria* belonging to the phylum Proteobacteria constituted 3–7% and 7–25% of total reads, respectively. As opposed to all other predominant phyla, Proteobacteria were represented by several dominating OTUs. *Alphaproteobacteria* were represented by *Rhodobacteraceae* with 1–2% of the total reads in all three samples, whereas *Gammaproteobacteria* were represented by the UBA10353 marine group (2–62% of *Gammaproteobacteria* and 0–6% of the total community), various *Enterobacterales* (4–69% of *Gammaproteobacteria* and 0–18% of the total community) with *Vibrio* sp. as the most numerous representative (22–44% of *Enterobacterales* and 0–9% of the total community) and the deep lineage SS1-B-07-19 (0–9% of *Gammaproteobacteria* and 0–3% of total community, depending on the specimen analyzed, Fig. 4). The Archaeal OTU identified in all samples as one of three dominating taxa was classified as *Candidatus* Nitrosopumilus (11–15% of the total community) belonging to the phylum Thaumarchaeota.

## Discussion

The *Tegulaherpia* sp., collected during this work is the first Solenogastres species found in underwater marine caves of the north-western Mediterranean. This species inhabits the upper layer of soft sediment. According to the content of the intestine (the presence of cnidarian nematocysts in the cells of the intestine), it was assumed that this species feeds on small burrowing Cnidaria, which were also found in this biotope (our own observations). The microscopic studies of all five specimens of *Tegulaherpia* sp. identified prokaryotes on the surface of the scales and through all the thickness of the cuticle. The microorganisms varied in morphology and localization: long rods and cocci were located on the surface of the cuticle while short rods and spirilla were inside the cuticle. The short rods were also numerous in the apical part of epithelial cells. This distribution of prokaryotes through the body of the mollusk is similar to that of the hot vent Solenogastres belonging to genus *Helicoradomenia* (Katz, Cavanaugh & Bright, 2006). Despite the fact that all studied individuals contained microorganisms and that microorganisms with different morphology were unequally distributed in the cuticle, we did not find evidences indicating physiological integration, such as would occur in specialized organs.

Association of Solenogastres and microorganisms has been poorly studied so far and based on the *Helicoradomenia* species living in deep-sea hydrothermal vents (Katz, Cavanaugh & Bright, 2006) and *Neomenia carinata* inhabiting soft sediments in moderate depths. However, in the latter case the only known fact is that bacteria are associated with the *Neomenia carinata* mantle epithelium (Scheltema, Tscherkassky & Kuzirian, 1994).

The microbial communities associated with *Tegulaherpia* sp*.,* detected by 16S rRNA gene amplicons sequencing, are dominated by prokaryotes of the bacterial phyla Bacteroidetes, Nitrospirae and Proteobacteria as well as the archaeal phylum Thaumarchaeota. Similarly to morphological observations, the microbial associates composition was consistent among the three individuals collected at different time, suggesting these associations were not artifactual. The presence of Thaumarchaeota and Bacteroidetes members makes these communities different from the *Helicoradomenia* sp. where these taxa were not detected (Katz, Cavanaugh & Bright, 2006). In the case of Thaumarchaeota this result seems to be solid since the FISH probe Arch915, used by *Katz* and co-authors in *2006*, is covering (86.1%) *Candidatus* Nitrosopumilus, what was verified using Silva TestProbe 3.0 (https://www.arb-silva.de/search/testprobe/). In the case of Bacteroidetes,the FISH probe CF319a does not cover (coverage = 1.5%) the *Saprospiraceae* family. On the other hand a large portion of cells of *Helicoradomenia* sp. symbionts were unknown bacteria (hybridized with universal bacterial primers but did not hybridize with any of the group-specific primer used by Katz, Cavanaugh & Bright (2006), suggesting at least some of them might be closely related to *Tegulaherpia* sp. bacterial symbionts including Bacteroidetes representatives.

The most abundant OTUs in *Tegulaherpia* sp. were related to *Saprospiraceae* (*Lewinella* and *Haliscomenobacter*), *Nitrospira* and *Candidatus* Nitrosopumilis. For each of them, symbiotic relationships with higher organisms are known. For instance, members of the genus *Lewinella*, were isolated from marine mollusks (sea snails) and were able to degrade polysaccharides and proteins (Khan et al., 2007). Their presence in the mollusks might indicate a symbiotic or parasitic relationship with *Tegulaherpia* sp. based on the degradation of cuticle polymers as chitin (Furuhashi et al., 2009) or glycoproteins with mucopolysaccharides (Beedham & Trueman, 1968). In turn, *Haliscomenobacter* representatives are widespread in a number of habitats including that of marine origin as marine waters, guts of marine fish (Gao et al., 2020), red algae healthy tissues (Fernandes et al., 2012) and others. Moreover, the dominating microorganisms in *Tegulaherpia* sp. microorganisms are known to form similar associations with other hosts: the community of a sponge, *Cymbastela concentrica*, was composed of the chemolithotrophic nitrite-oxidizing bacterium *Nitrospira* sp., a representative of the family *Phyllobacteriaceae* (*Alphaproteobacteria*) and the chemolithotrophic ammonia-oxidizing archaeon *Candidatus* Nitrosopumilus sediminis AR2 (Thaumarchaeota) (Moitinho-Silva et al., 2017). The sponge-microbial interaction might include production and sharing of various nutrients, *e.g.*, vitamins, as well as redox sensing and response. For instance, *Cymbastela concentrica* was supposed to contribute to the nitrogen metabolism of these microorganisms by supplying them with organic nitrogen compounds which they convert to ammonium, nitrite and nitrate during nitrification, denitrification and nitrate respiration (Moitinho-Silva et al., 2017).

In this respect the Thaumarchaeota and *Nitrospira* symbionts of *Tegulaherpia* sp. most probably contribute to a common nitrogen metabolism with their host whereas Bacteroidetes and *Alphaproteobacteria* and *Gammaroteobacteria* members are feeding on the mollusk‘s cuticle polymers or secreted compounds as it was proposed for *Helicoradomenia* sp. (Katz, Cavanaugh & Bright, 2006).

Altogether, our results document a novel Solenogastres species inhabiting environments so far unknown for these mollusks and possessing unique microbial associations on and within its cuticle. The observed microbial community is different from that found in *Helicoradomenia* species inhabiting deep sea hot vents. This is likely linked with the difference in the level of energy supply between these two habitats. Both are (almost) light-independent, however, unlike hot vents, underwater caves typically do not provide a constant inflow of reduced compounds needed for chemosynthesis, making cave species solely dependent on scarce organic compounds imported from the outside, which makes these caves similar to deep sea bottom sediments. Further studies are needed to reveal in more details the metabolic characteristics of the dominating microbial symbionts of *Tegulaherpia* sp. and their functional interactions with their host.

## Supplemental Information

10.7717/peerj.12655/supp-1 Supplemental Information 1 SSU taxonomic profiling results of SilvaNGS profiling of V4 16S rRNA gene amplicon sequences of Solenogastres microbial symbionts.

## Additional Information and Declarations

### Data Availability

The following information was supplied regarding data availability: The raw data are available in the Supplemental File.
